# Drug therapy problems among hospitalized patients with cardiovascular disease

**DOI:** 10.1186/s12872-024-03710-8

**Published:** 2024-01-15

**Authors:** Yirga Legesse Niriayo, Roba Kifle, Solomon Weldegebreal Asgedom, Kidu Gidey

**Affiliations:** Department of Clinical Pharmacy, School of Pharmacy, College of Health Sciences, Mekelle University, Mekelle, Tigray, Ethiopia

**Keywords:** Cardiovascular diseases, Drug therapy problems, Tigray

## Abstract

**Background:**

Optimal utilization of cardiovascular drugs is crucial in reducing morbidity and mortality associated with cardiovascular diseases. However, the effectiveness of these drugs can be compromised by drug therapy problems. Hospitalized patients with cardiovascular diseases, particularly those with multiple comorbidities, polypharmacy, and advanced age, are more susceptible to experiencing drug therapy problems. However, little is known about drug therapy problems and their contributing factors among patients with cardiovascular disease in our setting. Therefore, our study aimed to investigate drug therapy problems and their contributing factors in patients with cardiovascular diseases.

**Method:**

A prospective observational study was conducted among hospitalized patients with cardiovascular disease at Ayder Comprehensive Specialized Hospital in the Tigray region of Northern Ethiopia from December 2020 to May 2021. We collected the data through patient interviews and review of patients’ medical records. We employed Cipolle’s method to identify and categorize drug therapy problems and sought consensus from a panel of experts through review. Data analysis was performed using the Statistical Software Package SPSS version 22. Binary logistic regression analysis was performed to determine the contributing factors of drug therapy problems in patients with cardiovascular disease. Statistical significance was set at *p* < 0.05.

**Results:**

The study included a total of 222 patients, of whom 117 (52.7%) experienced one or more drug-related problems. We identified 177 drug therapy problems equating to 1.4 ± 0.7 drug therapy problems per patients. The most frequently identified DTP was the need for additional drug therapy (32.4%), followed by ineffective drug therapy (14%), and unnecessary drug therapy (13.1%). The predicting factors for drug therapy problems were old age (AOR: 3.97, 95%CI: 1.68–9.36) and number of medications ≥ 5 (AOR: 2.68, 95%CI: 1.47–5.11).

**Conclusion:**

More than half of the patients experienced drug therapy problems in our study. Old age and number of medications were the predicting factors of drug therapy problems. Therefore, greater attention and focus should be given to patients who are at risk of developing drug therapy problems.

## Introduction

Cardiovascular disease (CVD) is a major public health problem that affects about 523 million people worldwide [1, 2]. CVD is the leading cause of mortality on the globe, accounting for more than 17.9 million deaths per year, and it is projected to grow to more than 23.6 million by 2030 [3]. In Sub-Saharan Africa, CVDs are the most frequent cause of non-communicable disease related deaths, contributed to approximately 13% of all deaths and 38% of all non-communicable disease-related deaths [4, 5]. Likewise, the burden of CVD has been rising alarmingly in Ethiopia, and it is the leading cause of mortality [6]. Based on the global burden of the study (1990 to 2017), the age-standardized CVD prevalence, disability-adjusted life years and mortality rates in Ethiopia were 5534, 3549.6 and 182.63 per 100 000 population, respectively [7].

Despite the significant progress in the pharmacotherapy of CVDs in recent decades [8], they remain the leading cause of morbidity and mortality [9]. Although drug therapies play a crucial role in the management of illnesses including CVDs, they might have adverse outcomes unless utilized properly [10, 11]. In clinical practice, the treatment of CVDs remains challenging owing to the complexity of medication regimens, the availability of a wide range of drug products, multiple comorbidities, polypharmacy, advanced age, and lack of implementation of evidence based guidelines [11–14].

A drug therapy problem is any unfavorable event encountered by a patient that hinders the achievement of desired treatment goals through drug therapy, either in actuality or potentially [15]. DTPs can occur at various stages of the medication use process, starting from prescription processing to treatment follow-up [16]. Based on Cipolle’s method, DTPs are classified into seven major categories [15, 17]. These include; unnecessary drug therapy, the need for additional drug therapy, ineffective drug therapy, dosage too low, dosage too high, adverse drug reaction, and non-compliance [15, 17]. Globally, DTPs have significant social, economic, and humanistic impact [18–24]. From a social perspective, DTPs can undermine public confidence in healthcare systems and healthcare providers, which may lead to reluctance in seeking medical care [19, 20, 23]. Economically, DTPs result in increased healthcare costs due to hospital readmissions, prolonged treatments, decreased productivity, and legal expenses [21, 22, 25]. Moreover, at a humanistic level, these issues can inflict physical harm, suffering, and even loss of life, causing emotional distress for patients and their loved ones [23, 24].

Optimal use of cardiovascular drugs is crucial for reducing morbidity and mortality associated with CVDs. However, these benefits can be compromised due to DTPs [10, 26]. In patients with CVDs, the prevalence of DTPs ranged from 29.8 to 91% [10, 11, 27, 28]. Previous studies revealed that DTPs contributed to 28% of all admissions to emergency wards while 70-90% of them were potentially preventable [11]. Hospitalized patients with CVDs are at greater risk of developing DTPs owing to multiple comorbidities, polypharmacy, and old age [13, 28, 29].

In our current healthcare setting, there has been a lack of comprehensive investigation and documentation regarding DTPs among hospitalized patients with CVDs. To address this gap, it is necessary to conduct a study that can identify, quantify, and document these issues in CVD patients. This study would not only provide valuable insights into the extent of the problem but also shed light on existing gaps in healthcare practice. Moreover, it would increase awareness among healthcare professionals and policymakers, enabling them to focus on minimizing and preventing these problems. Therefore, this study aimed to investigate the magnitude of DTPs and their contributing factors in the management of patients with CVDs.

## Material and methods

### Study design and study setting

A prospective observational study was conducted among hospitalized patients with cardiovascular disease at Ayder Comprehensive Specialized Hospital in the Tigray region of Northern Ethiopia from December 2020 to May 2021. Ayder is a teaching and referral hospital that provides service for about 10 million people in the catchment area.

### Study participants

We enrolled adult patients (age > 18 years old) who were admitted to the medical ward with a diagnosis CVD. Patients with incomplete medical record and those who were unwilling to provide consent were excluded from the study. The sample size was calculated using a formula for estimating the sample size for a single population proportion [30, 31]. For populations with a size of ≥ 10,000, the formula is given as: n = [(Z1-α/2)² * p * (1-p)] / d². In this formula, n represents the minimum sample size, Z1-α/2 is the value at 95% confidence level (1.96), p was the estimated prevalence of DTP among patients with CVD (60.65%)) [11], and d is the margin of error to be tolerated (0.05). By substituting these values into the formula, we found that n was equal to 367. However, since the total population in our study was less than 10,000 (570), we recalculated the sample size using the correction formula: Nf = n / (1 + n/N), where Nf was the actual sample size using the correction formula, n was the minimum sample size (367), and N was the actual population size (570). By substituting these values into the formula, we found that Nf was equal to 224. Additionally, considering a 5% contingency for non-response rate, the minimum sample size required for this study was 235. Out of the 235 participants approached, a total of 13 patients were excluded from the study due to unwillingness to give consent [6] and incomplete medical records [7].

### Data collection procedure

We recruited patients upon admission to the medical ward using a simple random sampling technique. All patients were followed up daily until discharge. Data from each patient was collected daily to check for any changes in the treatment. Prior to participation, all individuals received a thorough explanation of the study’s objectives, and written informed consent was obtained from each participant. The data collection process involved patient interviews as well as expert reviews of patients’ medical, medication, and laboratory records. The responsibility of collecting the data was assigned to fifth-year clinical pharmacy students who were trained on the study’s objectives and methods of data collection.

### Assessment and identification of drug therapy problems

Drug therapy problems were first identified and categorized using Cipolle’s method [15], and then reviewed and validated by a panel of experts consisting of medical specialists and clinical pharmacists. Following this consensus review, the experts further refined the method for identifying and categorizing DTPs specifically for the study setting, taking into consideration treatment guidelines and literature reviews [32–34]. Specific information regarding medication therapies including the recommended drug of choice, recommended dosage regimens (dose of drug product, frequency of administration, and duration of therapy), drug-interactions and adverse drug events were compared based on details from the CVD’s treatment guidelines [33–41].

### Definition of terms and variables

In this study, individuals aged over 65 are considered to be in the old age category. Comorbidity refers to the presence of another medical condition alongside cardiovascular disease (CVD). Polypharmacy is defined as the simultaneous use of five or more medications [32]. A drug therapy problem is any unfavorable event encountered by a patient that hinders the achievement of desired treatment goals through drug therapy, either in actuality or potentially [15]. According to Cipolle’s method, DTPs are categorized into seven major classes, including unnecessary drug therapy, the need for additional drug therapy, ineffective drug therapy, dosage too low, adverse drug reaction, dosage too high, and noncompliance [15].

### Data analysis

Data were entered into EPI data management (version 4.2.0). Subsequently, data were exported in to the Statistical Package for Social Science (SPSS version 22.0) for analysis. Descriptive statistics were used to determine the frequency of categorical variables and the mean (standard deviation) of continuous variables. We checked multicollinearity among predictor variables using variance inflation factor (VIF) and none were collinear. We performed univariable logistic regression analysis to determine the association of each independent variable with DTPs. Subsequently, variables with *p* < 0.25 in univariable analysis were re-analyzed using a multivariable binary logistic regression model to identify predictors of DTPs. The statistical significance level was set at a *p*-value less than 0.05.

## Results

### Sociodemographic related characteristics

A total of 222 patients were included in the study. The mean age of the patients was 56.45 years, with a standard deviation of 17.76. Among the participants, 50.5% were male, 57.2% lived in urban areas, 69.4% were married, and 32.4% reported being unable to read and write (Table 1).

**Table 1:** Sociodemographic related characteristics of patients with CVD (*n* = 222)

Variables	n (%)
Gender, male	112(50.5)
Age in years	
15–35	38 (17.1)
36–65	107 (48.2)
> 65	77 (34.7)
Residence, urban	127(57.2)
Educational level,	
Unable to write and read	55 (24.8)
Primary education	25 (11.3)
Secondary education	87 (39.2)
College and above	55 (24.8)
Marital status,	
Married	154 (69.4)
Single	41 (18.5)
Divorced	12 (5.4)
Widowed	15 (6.8)
Alcohol	
No	159 (71.6)
Yes	63 (28.4)
Cigarette smoking	
No	203(91.4)
Yes	19(8.6)
Khat	
No	213(95.9)
Yes	9 (4.1)
Coffee	
No	44 (19.8)
Yes	178(80.2)

### Clinical related characteristics

Approximately 25% of the patients had one or more comorbidity. The mean (standard deviation) duration of hospital stay was 11.5 (10.2) days, and more than half (58%) of the patients were hospitalized for seven days or longer The most commonly diagnosed CVD was heart failure (45.3%), followed by hypertension (29%), and ischemic heart disease (26.12%) (Table 2).

**Table 2:** Clinical related characteristics of patients with CVD (*n* = 222)

Characteristics	n (%)
Duration of hospital stay	
≤ 7 days	93(42)
> 7 days	129(58)
Comorbidity	
No	165(74.3)
Yes	57 (25.7)
Commonly diagnosed CVDs	
Heart failure	102(45.3)
Hypertension	64(29)
Ischemic heart disease	58(26.12)
Stroke	40(18)
Atrial fibrillation	35(15.8)
Valvular heart disease	30(13.5)

*SD* Standard deviation, *CVD* Cardiovascular disease

### Treatment related characteristics

The mean (standard deviation) number of medications per patient was 3.43 (1.68), and one-third of the patients took five or more medications. The commonly prescribed medications included furosemide (43.7%), statins (36.9%), antiplatelets (33.3%), angiotensin converting enzyme inhibitors (27.5%), and beta blockers (23.9%) (Table 3).

**Table 3:** Treatment related characteristics of patients with CVD (*n* = 222)

Characteristics	n (%)
Number of medications	
< 5	149(67.1)
≥ 5	73 (32.9)
Frequently used medications	
Furosemide	97(43.7)
Statins	82(36.9)
Antiplatelets	74 (33.3)
Angiotensin converting enzyme inhibitors	61 (27.5)
Beta-blockers	53(23.9)
Calcium channel blockers	48(21.6)
Spironolactone	44 (19.8)
Anticoagulant	43(19.4)
Hydrochlorothiazide	18(8.1)
Digoxin	16(7.2)

### Prevalence of drug therapy problems

A total of 177 DTPs were detected, with a mean (standard deviation) of 1.4 (0.7) DTPs per patient. More than half (52.7%) of patients experienced one or more DTPs. The most frequently identified DTP was the need for additional drug therapy (32.4%), followed by ineffective drug therapy (14%) and unnecessary drug therapy (13.1%) (Table 4).

**Table 4:** Prevalence of DTPs among patients with CVD (*n* = 222)

Type of drug therapy problems	Causes of DTPS	Proportions of DTPs, n (%)
Unnecessary drugtherapy	No medical indication	16(7.2)
	Drug duplication	13(5.9)
Needs additional drug therapy	Untreated indication	55(24.8)
	Require additional pharmacotherapy to attain synergistic/additive effect	17(7.6)
Ineffective drug Therapy	Not the most effective among the available	25(11.3)
	Not effective for the condition	6(2.7)
Dosage too low	Dose too low to produce the desired response	11(4.9)
	Potential drug interaction causes reduced dose	2(1)
Adverse drug reaction	Undesirable reaction that was not dose-related	3(1.4)
	The drug is contraindicated due to risk factors	7(3.1)
Dosage too high	Dose too high	5 (2.3)
Non-adherence	Drug product was not available for the patient	6(2.7)
	Drug product was too expensive for the patient	4(1.8)
	Not remember to take the medication	3(1.4)
	The patient did not understand the instructions	2(0.9)
	Unwillingness to take the drug	2(0.9)

### Drugs commonly involved in drug therapy problems

In the study, the drugs most commonly implicated in DTPs were beta blockers (19.4%), followed by antithrombotics (14.4%), statins (13%), and angiotensin converting enzyme inhibitors (9%) as indicated in Table 5. These medications were frequently associated with the requirement for additional drug therapy. Moreover, beta blockers were frequently associated with ineffective treatment. Conversely, medications like calcium channel blockers (CCB), diuretics, and digoxin were found to be involved in unnecessary drug therapy.

**Table 5:** Drugs commonly involved in DTPs among patients with CVD (*n* = 222)

Drug class	n (%)
Beta blockers	43(19.4)
Anthithrombotics	32(14.4)
Statins	29(13)
Angiotensin converting enzyme inhibitors	19(9)
Loop diuretics	11(5)
Calcium channe blockers	11(5)
Potassium sparing diuretics drugs	9(3.6)
Cardiac glycosides	6(2.7)

### Factors associated with the drug therapy problems

The analysis using univariable logistic regression revealed that old age (Crude odds ratio [COR]: 3.44, 95% confidence interval [CI]: 1.52–7.74) and having five or more medications (COR: 2.66, 95% CI: 1.48–4.79) were significantly associated with the presence of DTPs. Subsequently, variables with a *p*-value less than 0.25 in the univariable analyses were included in the multivariable logistic regression model. The overall model, which included all predictors, showed statistical significance (Chi-square = 33.585, degrees of freedom = 7, *p* < 0.001). In the multivariate analysis, both old age (adjusted odds ratio [AOR]: 3.97, 95% CI: 1.68–9.36) and having five or more medications (AOR: 2.68, 95% CI: 1.47–5.11) remained significantly associated with DTPs (Table 6).

**Table 6:** Factors associated with drug therapy problems (*n* = 222)

Variables	DTP		COR (95% CI)	*P*-value	AOR (95%CI)	*P*-value
	No, n (%)	Yes, n (%)				
Sex						
Male	48(42.9)	64(57.1)	1		1	
Female	57(51.8)	53(48.2)	0.70(0.41–1.18)	0.182	0.84(0.47-1.49)	0.545
Age category						
18–35	22(57.9)	16(42.1)	1		1	
16–65	61(57.0)	46(43.0)	1.04(0.49–2.19)	0.924	1.14(0.52–2.49)	0.746
> 65	22(28.6)	55(71.4)	3.44(1.52–7.74)	0.003	3.97(1.68–9.36)	0.002
Residence						
Urban	54(42.5)	73 (57.5)	1		1	
Rural	51(53.7)	44(46.3)	0.64(0.37–1.09)	0.100	0.59(0.32–1.07)	0.083
Duration of Hospitalization r						
≤ 7 days	43(42.2)	59(57.8)	1		1	
> 7 days	62(51.7)	58(48.3)	0.68(0.40–1.16)	0.158	0.82(0.46–1.48)	0.518
Comorbidity						
No	82(49.7)	83(50.3)	1		1	
Yes	23(40.4)	34(59.6)	1.46(0.79–2.69)	0.224	1.28(0.64–2.58)	0.482
Number of medications						
< 5	82(55)	67(45)	1		1	
>=5	23(31.5)	50(68.5	2.66(1.48–4.79)	0.001	2.68(1.47–5.11)	0.003

* COR* Crude odds ratio, *AOR* Adjusted odds ratio, *CI* Confidence interval

## Discussion

Despite the fact that the majority of drug therapy problems (DTPs) can be prevented, they remain a major healthcare challenges in clinical practice [11, 32]. DTPs can result in increased morbidity, reduced quality of life, increased health care costs, and even death if not identified and resolved promptly [32]. It is essential to evaluate DTPs and understand the factors contributing to their occurrence in CVDs to develop effective intervention programs for the future. Therefore, the aim of our study was to investigate DTPs and their contributors among hospitalized patients with CVDs.

Our study revealed that more than half (52.7%) of the patients experienced one or more DTPs despite the fact that DTPs have been associated with negative outcomes in CVDs [10]. In agreement with our study, comparable findings were reported in previous studies conducted in Saudi Arabia and Ethiopia [11, 42, 43]. This suggests that the problem is not isolated to a specific geographic location but rather a widespread issue that needs attention. In contrast, our study found a higher incidence of DTPs compared to a study conducted in Spain, where the reported rate was 29.8%) [28]. This discrepancy may be attributed to several factors commonly observed in developing countries like Ethiopia, including the low level of health literacy, the absence of well-defined protocols, poor belief in modern medicine, poor health care system, and inadequate supply of cardiovascular drugs [44–46]. It is important to address these challenges in developing countries to optimize cardiovascular care and reduce the prevalence of DTPs.

Among the identified DTPs, the most frequent was the need for additional drug therapy, followed by ineffective drug therapy and unnecessary drug therapy. Similarly, the need for additional drug therapy was the most frequently identified DTP in previous studies conducted in Ethiopia [11, 27]. This observation can be attributed to a variety of factors, including complex medical conditions necessitating multiple medications, presence of comorbidities, missed diagnoses, and adverse drug events. Additionally, socio-economic factors like limited healthcare access or affordability issues, medication non-adherence, individual variations in medication response, and evolving diseases may contribute to the need for additional drug therapy. Effective communication between healthcare providers and patients, as well as proper patient education and comprehensive healthcare strategies are important to minimize this DTP and optimize patient care. Consistent with our findings, ineffective drug therapy and unnecessary drug therapy were among the frequently identified DTPs in other similar studies [32, 42]. In contrast, adverse drug reaction was the most common DTP in Cyprus study [47]. This variation may be attributed to differences in identifying and classifying DTPs, healthcare infrastructure and practices, as well as population demographics.

In the current study, beta blockers (19.4%), antithrombotics (14.4%), statins (13%), and angiotensin-converting enzyme inhibitors (9%) were the most commonly implicated classes of drugs in DTPs. This finding is consistent with a study conducted in Jimma, Ethiopia among hospitalized heart failure patients, where beta blockers (35%), angiotensin-converting enzyme inhibitors (25%), antithrombotics (20%), and statins (16%) were frequently implicated in DTPs [43]. Similarly, a study on ambulatory cardiac patients in Jimma reported similar findings [32]. Despite evidence-based guidelines recommending the use of beta blockers, statins, antiplatelets, and angiotensin-converting enzyme inhibitors in all cases of acute coronary syndrome, unless contraindicated [48, 49], a considerable number of patients did not receive these medications, despite their necessity in our study. Moreover, even though beta blockers are recommended for all cases of systolic heart failure in the absence of contraindications [50], heart failure patients were not receiving beta blockers despite their need for them. Furthermore, it was observed that atenolol, which is not an approved drug for heart failure in clinical trials, was commonly used instead of the approved beta blockers carvdeilol, bisoprolol, and metoprolol [50]. Moreover, beta blockers were sometimes inappropriately used as monotherapy for hypertension, despite not being first-line treatments [33]. These concerning issues can be attributed to the lack of comprehensive CVD treatment guidelines in our setting. The development and implementation of such guidelines would assist healthcare providers in selecting the appropriate medications for their patients, thereby ensuring optimal treatment outcomes and reducing the occurrence of DTPs.

Polypharmacy has consistently been identified as a major contributing factor to DTPs in multiple studies [13, 28, 51, 52]. Correspondingly, the current study found a significant association between the number of medications and DTPs. Specifically, our study revealed that patients who took five or more drugs were about three times more likely to experience DTPs compared to those with a smaller number of medications. Supporting this observation, a similar local study conducted in Jimma, Ethiopia reported consistent findings [43]. Polypharmacy can lead to drug therapy problems because it increases the risk of adverse drug reactions, drug interactions, medication non-adherence, and medication errors [32, 53, 54]. Furthermore, polypharmacy can also complicate medication management, especially for elderly patients with multiple chronic conditions [55, 56]. Hence, it becomes crucial to regularly evaluate and optimize medication regimens in order to effectively address the challenges associated with polypharmacy.

Furthermore, age was another significant factor associated with DTPs in this study. Elderly individuals (aged > 65 years) were four times more likely to experience DTP compared to younger adults (aged 18–35 years). This finding was also supported by other studies [29, 32]. The rationale behind this observation lies in the fact that older patients often have multiple comorbidities, along with renal and liver impairments, and are likely to be on multiple medications [57, 58]. Consequently, the elderly population becomes more vulnerable to dosing errors, adverse drug effects, drug interactions, and non-compliance [59, 60]. Age-related cognitive decline can also impact medication adherence and proper use [61, 62]. Therefore, considering age-related factors is crucial when evaluating drug therapy to ensure safe and effective treatment for older patients.

### Limitation of the study

Although we made efforts to consider several factors that could potentially affect DTPs, we did not specifically evaluate the influence of healthcare professionals’ level of knowledge on DTPs. Moreover, as this study’s findings could be influenced by disparities in population demographics, disease prevalence, healthcare systems, qualifications of healthcare providers, and methodologies utilized, it is crucial to exercise caution when extrapolating these results to other countries.

## Conclusion

Our study revealed that more than half of the patients have experienced DTPs. Old age and polypharmacy were identified as significant predictors of DTP. Therefore, more emphasis should be given to patients at risk of developing drug therapy problems. More importantly, substantial efforts should be made to mitigate the potentially modifiable risk factors associated with DTPs in the treatment of CVD. Measures such as implementing medication reconciliation and standardized clinical practices have the potential to effectively reduce the occurrence of DTPs among patients with CVDs.

## Data Availability

The dataset of this article is accessible on reasonable request from the corresponding author.

## Abbreviations

AOR: Adjusted Odds Ratio
ADR: Adverse Drug Reaction
CI: Confidence Interval
COR: Crude Odds Ratio
CVD: Cardiovascular Disease
DTP: Drug Therapy Problem
SD: Standard Deviation
SPSS: Statistical Package for Social Science
